# Connectivity from OR37 expressing olfactory sensory neurons to distinct cell types in the hypothalamus

**DOI:** 10.3389/fncir.2012.00084

**Published:** 2012-11-16

**Authors:** Andrea Bader, Bettina Klein, Heinz Breer, Jörg Strotmann

**Affiliations:** Institute of Physiology, University of HohenheimStuttgart, Germany

**Keywords:** olfaction, OR37, paraventricular nucleus, supraoptic nucleus, vasopressin, wiring

## Abstract

Olfactory sensory neurons (OSNs) which express a member from the OR37 subfamily of odorant receptor (OR) genes are wired to the main olfactory bulb (MOB) in a unique monoglomerular fashion; from these glomeruli an untypical connectivity into higher brain centers exists. In the present study we have investigated by DiI and transsynaptic tracing approaches how the connection pattern from these glomeruli into distinct hypothalamic nuclei is organized. The application of DiI onto the ventral domain of the bulb which harbors the OR37 glomeruli resulted in the labeling of fibers within the paraventricular nucleus (PVN) and supraoptic nucleus (SO) of the hypothalamus; some of these fibers were covered with varicose-like structures. No DiI-labeled cell somata were detectable in these nuclei. The data indicate that projection neurons which originate in the OR37 region of the MOB form direct connections into these nuclei. The cells that were labeled by the transsynaptic tracer WGA in these nuclei were further characterized. Their distribution pattern in the paraventricular nucleus was reminiscent of cells which produce distinct neuropeptides. Double labeling experiments confirmed that they contained vasopressin, but not the related neuropeptide oxytocin. Morphological analysis revealed that they comprise of magno- and parvocellular cells. A comparative investigation of the WGA-positive cells in the SO demonstrated that these were vasopressin-positive, as well, whereas oxytocin-producing cells of this nucleus also contained no transsynaptic tracer. Together, the data demonstrates a connectivity from OR37 expressing sensory neurons to distinct hypothalamic neurons with the same neuropeptide content.

## Introduction

Numerous vital behaviors such as the finding of food sources, avoiding predators, and recognizing conspecifics are triggered by olfactory cues. In mammalian species, the relevant odorous molecules are detected by specialized olfactory sensory neurons (OSNs) which are organized in structurally and functionally distinct chemosensory subsystems [for reviews see (Breer et al., 2006; Ma, 2007; Munger et al., 2009)]. One special subsystem is located within the main olfactory system (MOS) and is formed by the so-called OR37 subfamily of odorant receptors (ORs) (Strotmann et al., 1992). This type of OR is structurally unique due to an insertion of six amino acids in its third extracellular loop (Kubick et al., 1997); furthermore, it exists exclusively in mammalian species (Hoppe et al., 2006), and against the general trend for ORs, the various OR37 subtypes are highly conserved among each other and across species borders from opossum to human (Hoppe et al., 2006). These features have led to the speculation that the OR37 subsystem may be tuned to detect special chemical compounds, which might be particularly relevant for mammals. In fact, we have recently demonstrated that the OR37 receptors are specifically activated by long-chain fatty aldehydes (Bautze et al., 2012); these compounds are extremely hydrophobic and were never described as active ligands for any other ORs in previous studies.

These aspects raise the question whether the information received via the OR37 sensory cells may also be processed in the brain in a way that differs from that of general OSNs. Indeed, the visualization of axonal projections from OSNs expressing defined OR37 receptors into the main olfactory bulb (MOB) has revealed that they target only a single glomerulus (Strotmann et al., 2000), a pattern which is clearly different from that of most other OSN populations which typically send their axons to two glomeruli [for recent reviews see (Imai et al., 2010; Sakano, 2010; Mori and Sakano, 2011; Murthy, 2011)]. Interestingly, the glomeruli for the different OR37 subtypes were found to be grouped together in the ventral domain of the MOB, a region which is believed to play a role in the processing of socially relevant olfactory cues (Schaefer et al., 2001, 2002; Lin et al., 2007). Using DiI and a genetic tracing approach we have recently provided first data showing that also the connectivity from a defined OR37 glomerulus into higher brain centers is organized in a special manner (Bader et al., 2012). In contrast to the prevailing principle that projection neurons from the MOB, the mitral/tufted (M/T) cells, send their axons to the piriform cortex, the anterior olfactory nucleus or the cortical amygdala which together form the olfactory cortex, a connectivity between an OR37 glomerulus and the medial amygdala (Me) was discovered. This finding is particularly notable since the Me receives input primarily from a different chemosensory subsystem, the accessory olfactory system (Kevetter and Winans, 1981) which is involved in the processing of pheromonal information (Halpern and Martinez-Marcos, 2003). The connectivity from the OR37 glomerulus was found to be confined to even a particular subregion of the Me, namely the posterior-dorsal subnucleus (MePD). These data thus provided first evidence that the projection from the OR37 subsystem into higher brain centers is in fact different from the general design of the MOS.

Our data have indicated that in addition to the special relay into the amygdaloid complex, a connection between the OR37 glomerulus and distinct hypothalamic nuclei, the supraoptic nucleus (SO), and the paraventricular nucleus (PVN) seems to exist. It remained unclear in these cases, however, whether the connectivity into these regions is made up directly by the axons of projection neurons originating from an OR37 glomerulus, or indirectly via for example the amygdala. Furthermore, toward an insight into the functional relevance of this connection, the identification of the cell type(s) to which it is formed, is an essential step. It was therefore the aim of the present study to further characterize the type of connectivity between the OR37 system and these particular hypothalamic nuclei.

## Materials and methods

### Experimental animals

All procedures were approved by the local authorities. Animals were kept at the University of Hohenheim transgenic core facility; housing conditions fulfil the animal welfare guidelines. Two different mouse strains were used carrying a targeted mutation of IRES-tauGFP either at the OR37C-locus or at the OR37A-locus (short: OR37-ITGFP) (Strotmann et al., 2000). Additionally the previously generated mouselines were used that carry an OR37C-IRES-WGA transgene (Bader et al., 2012). For tissue preparations adult animals (minimum 8 weeks) were killed by cervical dislocation and subsequent decapitation as approved by the regional administrative authority (Regierungspräsidium Stuttgart # S136/02 Phy).

### Neuronal tracer application

After decapitation of OR37-ITGFP mice the bones covering the dorsal aspects of the brain and the tip of the nose were removed. Subsequently the palatal bones were split just behind the most caudal teeth and the nasal turbinates were carefully separated from the olfactory bulb with a forceps. Thereby the ventral surface of the olfactory bulb was exposed. In OR37-ITGFP mice the glomeruli innervated by OR37A− or OR37C-expressing neurons, respectively, could be identified by their bright GFP-fluorescence that was visualized using a stereomicroscope (SterREO Lumar.V12; Zeiss) equipped with appropriate filters. A microneedle was used to place a small DiI crystal (1,1′-dioctadecyl-3,3,3′,3′-tetramethylindocarbocyanine perchlorate; Molecular Probes) precisely onto these glomeruli. The extent of the application site was controlled by visualizing the fluorescence of the DiI crystal with appropriate filters. Immediately after tracer application, specimens were placed in 4% paraformaldehyde (in 150 mM phosphate buffer, pH 7.4) and incubated overnight at 4°C. Subsequent incubation was performed at 37°C in the dark for several (12 or 26) weeks. Afterwards the bones covering the ventral side of the brain were removed. Finally, 4% agarose (Genaxxon) was melted in 1× phosphate buffered saline (PBS) (0.85% NaCl, 1.4 mM KH_2_PO_4_, 8 mM Na_2_HPO_4_, pH 7.4) and brain tissue was embedded in this substrate. After polymerization of the agarose the specimens were prepared for subsequent sectioning.

### Sectioning of neuronal tracer specimens

Free-floating sections (60 μm) were generated using a VT1000S vibrating microtome (Leica Microsystems). Consecutive sections were transferred to individual cups of 24-well plates supplied with 1× PBS and residual agarose was removed. Every other section was immediately mounted onto “Star Frost” microscope slides (Knittel Gläser), covered with MOWIOL (13% Mowiol 4-88, 33% Glycerin, 130 mM Tris, pH 8.5) and a coverslip (Menzel Gläser). The corresponding sections were used for immunohistochemical stainings.

### Immunohistochemistry on free-floating sections

For labeling of vasopressinergic neurons on free-floating sections, mouse anti-neurophysin-vasopressin antibody (PS41; kindly provided by Dr. Harold Gainer; NIH; Bethesda; USA) was diluted 1:100 in 0.3% Tween-20/ 1× PBS containing 10% normal donkey serum (NDS; Jackson ImmunoResearch). Sections were incubated with the diluted primary antibody overnight at 4°C. After three rinses for 5 min in 1× PBS, the bound primary antibodies were visualized using appropriate secondary antibodies conjugated to Alexa 488 (Invitrogen) diluted in 0.3% Tween-20/ 1× PBS containing 10% NDS for 2 h at room temperature. After washing three times for 5 min in 1× PBS the sections were rinsed with distilled water, transferred onto “Star Frost” microscope slides (Knittel Gläser) and finally mounted in MOWIOL.

### Tissue preparation for cryosectioning

After decapitation all bones of the head surrounding the brain and the nasal turbinates were excised. The specimens were fixed by immersion in 4% paraformaldehyde (in 150 mM phosphate buffer, pH 7.4) for 1 and 4 h on ice. Subsequently the tissue was cryoprotected by incubation in 25% sucrose/ 1× PBS overnight at 4°C. Finally, the tissue was embedded in “Tissue Freezing Medium” (Leica Microsystems) and frozen on dry ice or liquid nitrogen.

### Cryosectioning

Cryosections (10 to 12 μm) were generated using a CM3050S cryostat (Leica Microsystems) and mounted onto “SuperFrost” microscope slides (Menzel Gläser).

### Nissl staining

After drying cryosections for approximately 1 h at 37°C, the slides were transferred to a glass container containing 1:1 chloroform/ethanol and incubated over night at room temperature. Subsequently sections were rehydrated via a series of ethanol dilutions (5 min in 100% ethanol; 5 min in 95% ethanol; 5 min in 70% ethanol) followed by a final incubation in distilled water. Staining was performed for 15 min in 0.1% Cresyl Violet at 55°C. Residual dye was removed via a short rinse in distilled water. Subsequently sections were again dehydrated (3 min in 95% ethanol; 10 min in 100% ethanol) and tissue was cleared with incubation in Xylol for 10 min. Finally sections were air dried and mounted in Vectamount mounting medium (Vector Laboratories).

### Immunohistochemistry

For immunohistochemical experiments cryosections were air dried and rinsed in 1× PBS for 10 min. For single and double labeling experiments primary antibodies [mouse anti-neurophysin-vasopressin antibody (PS41) 1:50; rabbit anti-vasopressin (VA4) 1:300; rabbit anti-oxytocin (VA10) 1:1000 (all kindly provided by Dr. Harold Gainer; NIH; Bethesda; USA); goat anti-WGA 1:300 (Vector Laboratories)] were diluted in 0.3% Triton X-100/1× PBS containing 10% NDS. Sections were incubated with the diluted primary antibodies overnight at 4°C. After three rinses for 5 min in 1× PBS, the bound primary antibodies were visualized using appropriate secondary antibodies conjugated to Alexa 488 (Invitrogen) or Cy3 (Jackson ImmunoResearch) diluted in 0.3% Triton X-100/1× PBS containing 10% NDS for 2 h at room temperature. After washing three times for 5 min in 1× PBS the sections were rinsed with distilled water and finally mounted in MOWIOL.

### Analysis, microscopy, and photography

Whole mount preparations of the olfactory bulb were analyzed using a SterREO Lumar.V12 (Zeiss) equipped with appropriate filters. Images were captured using a “Sensi-Cam” CCD-camera (PCO-imaging). Free-floating sections were analyzed for DiI-fluorescence and immunofluorescence on consecutive sections, respectively, using an inverted confocal laser scanning microscope (LSM 510 Meta; Zeiss) equipped with a Plan-Neofluar 10×/0.3 objective (Zeiss) and a C-Apochromat 63×/1.2 W corr. objective (Zeiss); digital images were processed in the Zeiss AIM 510 software and represent projections of Z-stacks. Cryosections were analyzed using an Axiophot microscope (Zeiss) equipped with Plan-Neofluar 10×/0.3, Plan-Neofluar 20×/0.5 and Plan-Neofluar 40×/0.75 objectives (Zeiss); images were captured using an Axiocam (Zeiss) for transmitted light and a “Sensi-Cam” CCD-camera (PCO-imaging) for fluorescent images. Pictures were arranged using PowerPoint (Microsoft). Brightness and contrast were adjusted for the whole images. Morphological structures in the mouse brain were identified according to the mouse brain atlas of Paxinos and Franklin (2001).

## Results

**Figure 1:**
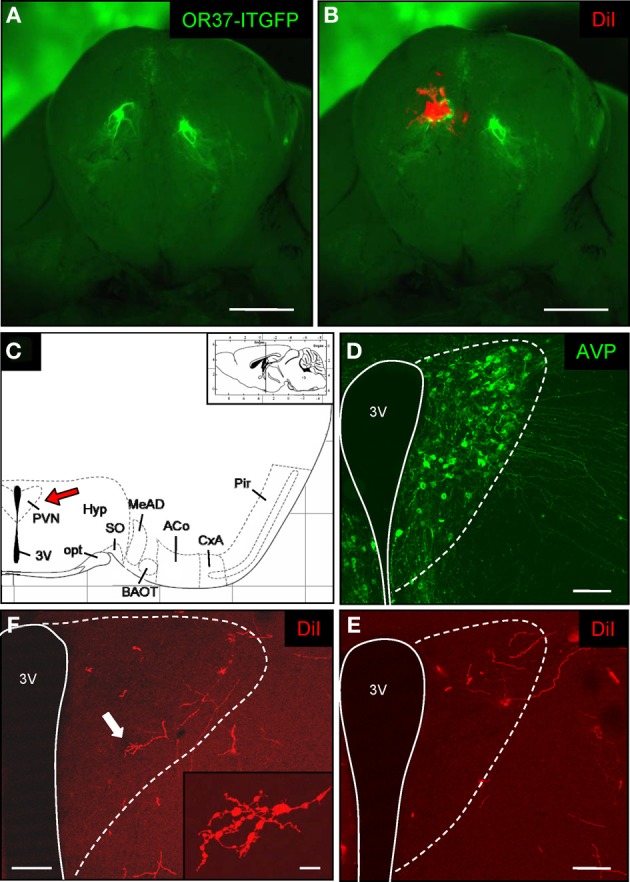
**DiI-labeling in the paraventricular nucleus of the hypothalamus after tracer application onto the OR37 glomerulus. (A)** Whole-mount preparation of the olfactory bulbs from an OR37-ITGFP mouse. A single fluorescent glomerulus (green) is visible on the ventral surface of each bulb. Scale bar: 1 mm. **(B)** A DiI crystal (red) was deposited on the fluorescent glomerulus. Scale bar: 1 mm. **(C)** Schematic representation of a section through the mouse brain [adapted from Paxinos and Franklin (2001)]. The position of the section along the anterior–posterior axis is indicated on the inset. The interval of the lattice is 1 mm. Several distinct nuclei are indicated. The red arrow highlights the paraventricular nucleus. 3V, third ventricle; ACo, anterior cortical amygdala; BAOT, bed nucleus of the accessory olfactory tract; CxA, cortex-amygdala transition zone; MeAD, medial amygdala anterior-dorsal part; opt, optic tract; Pir, piriform cortex; PVN, paraventricular nucleus; SO, supraoptic nucleus. **(D)** Confocal image of a tissue section through the paraventricular nucleus. Vasopressinergic cells are stained with a specific antibody (anti-neurophysin-vasopressin). According to this cell population the extent of the nucleus close to the third ventricle (3V) can be estimated (dotted lines) and is transferred to the neighboring section shown in **(C)**. Pinhole size: 70 μm. Scale bar: 100 μm. **(E)** On the neighboring section distinct DiI-labeled fibers are located in the paraventricular nucleus after application of a DiI-crystal onto the OR37 glomerulus and subsequent incubation for 12 weeks. Pinhole size: 80 μm. Scale bar: 100 μm. **(F)** DiI-labeled fibers in the paraventricular nucleus of an individual 26 weeks after dye application. The arrow highlights the fiber shown on the inset. At high magnification, multiple ramifications and varicose-like swellings along the fiber are visible. Pinhole size: 80 μm; inset: 87 μm. Scale bar: 100 μm; inset: 10 μm.

To address the question whether a direct cellular connectivity from an OR37 glomerulus into the PVN of the hypothalamus may exist, we exploited the fact that the lipophilic dye molecule DiI, when inserted into the plasma membrane of a given cell, spreads by lateral diffusion within the processes of this particular cell; such an approach has been used in previous studies to unravel the direct connectivity between defined brain regions (Godement et al., 1987; Honig and Hume, 1989). The fluorescently labeled OR37 glomerulus in our OR37-ITGFP transgenic mouse lines (Figure 1A) (Strotmann et al., 2000) served as a landmark for the deposition of a dye crystal (Figure 1B), anticipating that the dye would get into contact with the dendrites of projection neurons which are associated with OR37 glomeruli. After an appropriate incubation time of the tissue, serial sections through the hypothalamus were analyzed. The PVN can be recognized due to its position in close vicinity to the third ventricle (Figure 1C); however, for an unambiguous identification we used the fact that it contains, among many others, arginine-vasopressin (AVP)-producing neurons (Kadar et al., 2010; Biag et al., 2012). Immunohistochemical analyses of serial sections through the hypothalamus in fact revealed AVP-positive cells within a well circumscribed region close to the third ventricle (Figure 1D), thereby delineating the nucleus. Every other section was examined for the presence of DiI-fluorescence. Close inspections revealed DiI-labeled filamentous structures within the region of the AVP-positive cells (Figure 1E); they were sparsely dispersed throughout the corresponding region. After a longer incubation time, a picture as representatively shown in Figure 1F emerged. At high magnification, ramifications of the fiber-like structures with small terminal-like endings were visible; many of them were covered with varicose-like elements, indicating a possible *en passant* synaptic arrangement with cells within the PVN. The application of DiI onto the OR37 glomerulus in the MOB thus clearly labeled fibers in this particular hypothalamic nucleus (*n* = 6); a close examination of the nucleus revealed no staining of cell bodies. Together these results support the concept that a direct connectivity between the OR37 region of MOB and this hypothalamic nucleus exists which is formed by the axonal processes of projections neurons which are situated at the site of dye application at the OR37 glomeruli.

**Figure 2:**
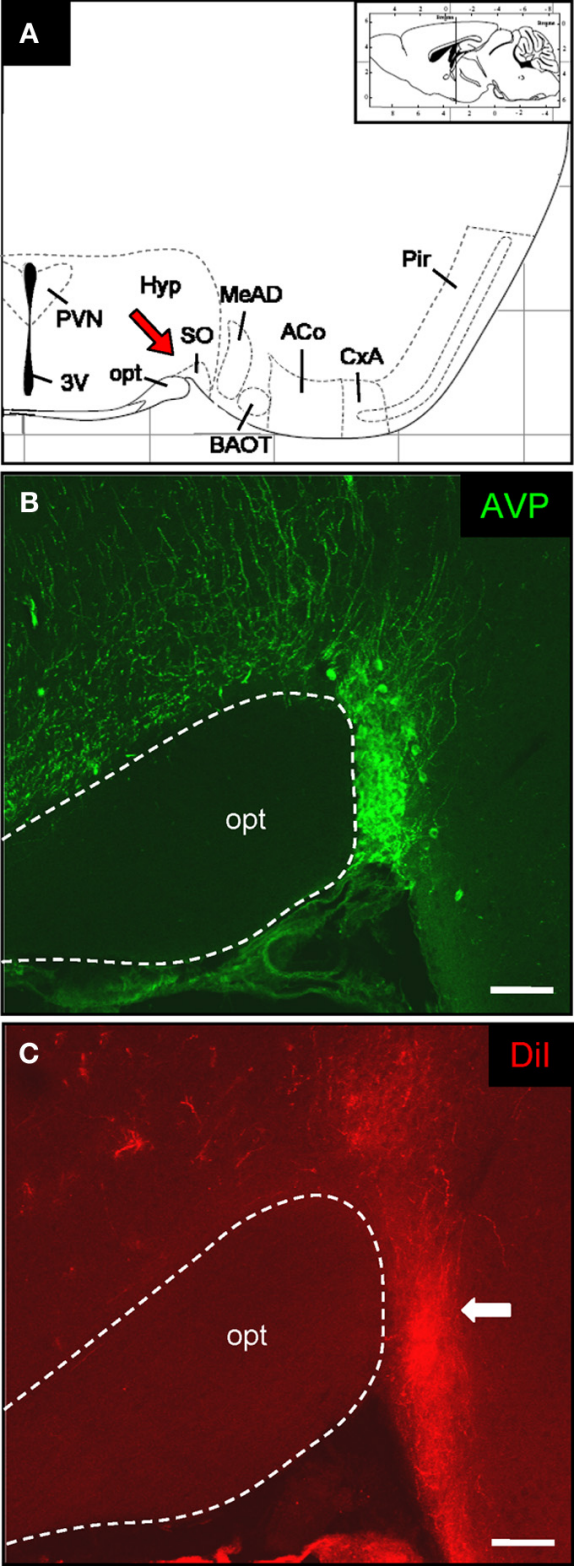
**DiI-labeled fibers in the supraoptic nucleus of the hypothalamus. (A)** Schematic representation of a section through the mouse brain [adapted from Paxinos and Franklin (2001)]. The position of the section along the anterior–posterior axis is indicated on the inset. The interval of the lattice is 1 mm. Several distinct nuclei are indicated. The red arrow highlights the supraoptic nucleus. 3V, third ventricle; ACo, anterior cortical amygdala; BAOT, bed nucleus of the accessory olfactory tract; CxA, cortex-amygdala transition zone; MeAD: medial amygdala anterior-dorsal part; opt, optic tract; Pir, piriform cortex; PVN, paraventricular nucleus; SO: supraoptic nucleus. **(B)** Confocal image of a cross section through the supraoptic nucleus. Vasopressinergic cells are stained with a specific antibody (anti-neurophysin-vasopressin). According to this cell population the position of this particular hypothalamic nucleus close to the optic tract can be exactly determined. Pinhole size: 70 μm. Scale bar: 100 μm. **(C)** After application of DiI onto the OR37 glomerulus a prominent bundle of labeled fibers can be visualized on the neighboring tissue section. Comparing the two staining patterns shows that the DiI-labeled fibers extend up to the somatic portion of the supraoptic nucleus (arrow). Pinhole size: 80 μm. Scale bar: 100 μm.

To examine whether OR37 M/T-cells also project directly to the SO, we examined this region after placement of the DiI-crystal onto the fluorescent OR37 glomerulus. The SO can be found at the ventral base of the brain in immediate vicinity to the optic tract (see Figure 2A). Also in this case, we made use of the fact that it contains AVP-producing neurons to unequivocally identify it (Ludwig et al., 2005). By using an immunohistochemical approach, a densely packed group of AVP-positive cell bodies could be visualized in neighborhood to the optic tract (Figure 2B). Consecutive sections were examined for the presence of the dye. In contrast to the rather sparse staining in the PVN, a thicker bundle of DiI-positive fibers was visible extending to the portion of the SO where the corresponding cell somata are located (Figure 2C) (*n* = 6). These data thus provided evidence that also the SO of the hypothalamus receives direct input from M/T-cells originating at the OR37 region of the MOB.

**Figure 3:**
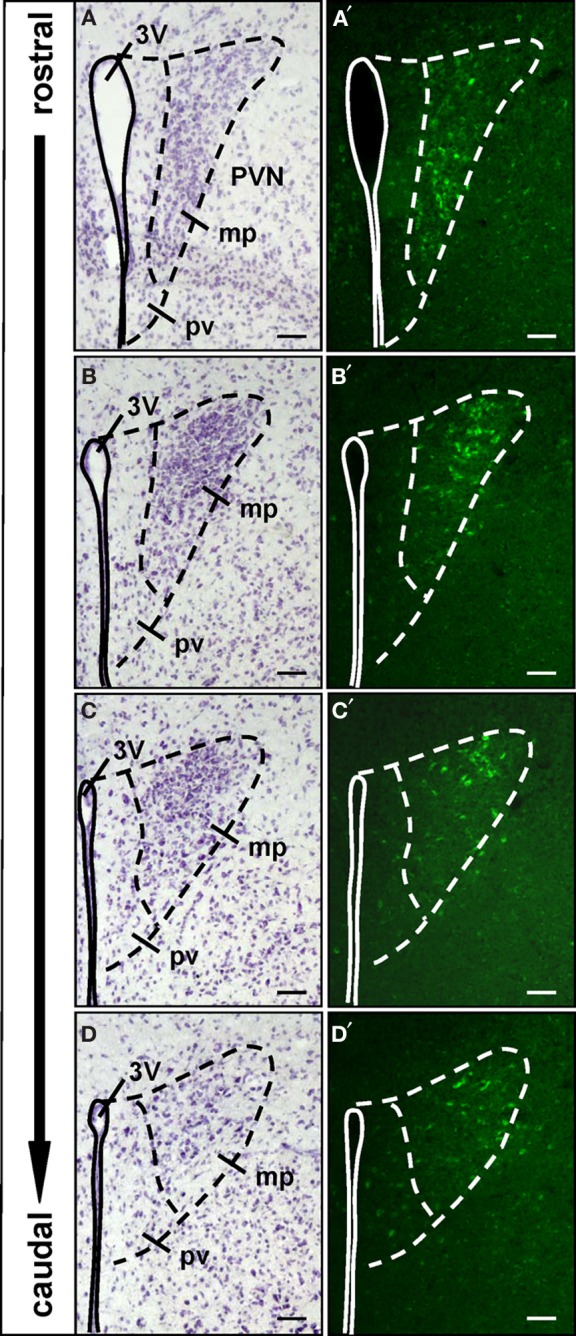
**Spatial organization of the WGA-immunoreactive cells in the paraventricular nucleus. (A–D)** Nissl-stained cross sections through the paraventricular nucleus (PVN). The third ventricle (3V) is indicated by the continuous line. The dashed lines mark the extent of the paraventricular nucleus and separate the periventricular part (pv) from the major portion (mp) of the nucleus. These divisions are transferred to the respective neighboring sections shown in **(A′–D′)**. **(A′–D′)** All along the anterior–posterior axis of the paraventricular nucleus, no WGA-immunoreactivity can be observed in the periventricular part. WGA-immunoreactive cells are located in the major portion of the nucleus. In the rostral part these cells are clustered in the ventromedial subdivision, at more caudal levels the WGA-immunoreactivity is predominantly located in dorsolateral parts. All pictures represent wide field images. The spacing between the sections from different areas along the anterior-posterior axis is approximately 60–96 μm, respectively. Scale bars: 50 μm.

**Figure 4:**
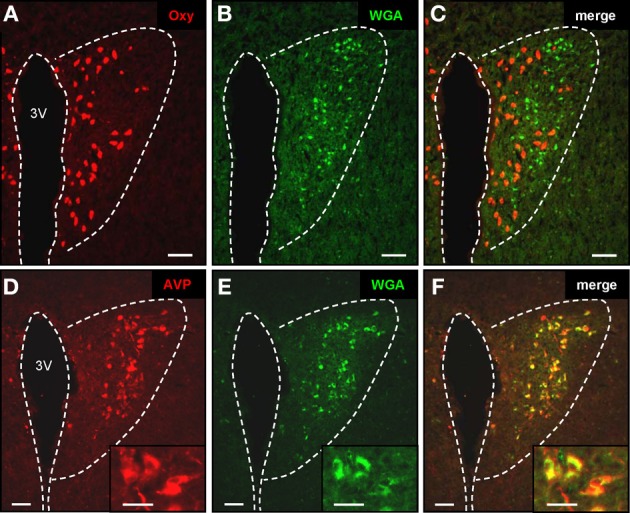
**Transsynaptic labeling of vasopressinergic neurons in the paraventricular nucleus. (A–C)** Cross section through the paraventricular nucleus. By means of a specific anti-oxytocin antibody, multiple oxytonergic neurons can be visualized in close vicinity to the third ventricle (3V) (**A**; red). On the same section WGA-immunoreactive cells can be observed (**B**; green). Merging the two pictures **(C)** reveals that oxytonergic neurons are not labeled by the transsynaptic marker. Scale bars: 50 μm. **(D–F)** Tissue section through the paraventricular nucleus. After staining with an anti-vasopressin antibody, neurons are visible near the third ventricle (3V) (**A**; red). WGA-immunoreactive cells are located on the same section (**B**; green). Merge of pictures **(A)** and **(B)** reveals that vasopressinergic cells are labeled by the transsynaptic marker (**C**; yellow). Scale bars: 50 μm. Higher magnifications are shown on the insets. Scale bars: 25 μm. All pictures represent wide field images.

**Figure 5:**
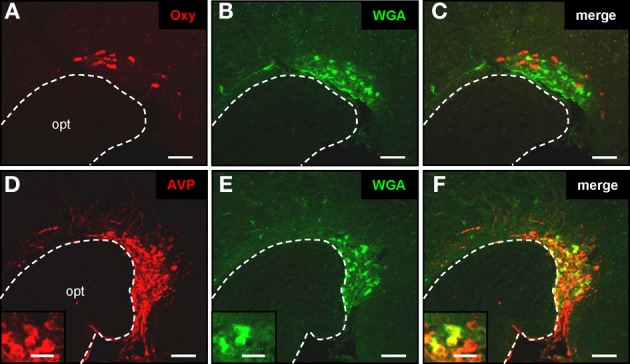
**Transsynaptic labeling of vasopressinergic neurons in the supraoptic nucleus. (A–C)** Cross section through the supraoptic nucleus. By means of a specific anti-oxytocin antibody, multiple oxytonergic neurons can be visualized near the optic tract (opt) (**A**; red). On the same section WGA-immunoreactive cells can be observed (**B**; green). Merging the two pictures **(C)** reveals that oxytonergic neurons are not labeled by the transsynaptic marker. Scale bars: 50 μm. **(D–F)** Tissue section through the supraoptic nucleus. After immunohistochemical staining with an anti-neurophysin-vasopressin antibody, neurons are visible in close vicinity to the optic tract (opt) (**A**; red). On the same section WGA-immunoreactive cells are located (**B**; green). Merge of pictures **(A)** and **(B)** reveals that vasopressinergic cells are labeled by the transsynaptic marker (**C**; yellow). Scale bars: 50 μm. Higher magnifications are shown on the insets. Scale bars: 20 μm. All pictures represent wide field images.

Regarding the functional role of the relays between the OR37 region of the MOB and these hypothalamic nuclei, the knowledge about the cell type(s) to which this connection is formed, is an essential step. To address the question we used our previously generated transgenic mouse lines in which OR37C expressing sensory neurons coexpress the transsynaptic marker WGA (Bader et al., 2012); in these transgenic mice the cells in the hypothalamus to which this marker is transferred can be identified by their WGA-immunoreactivity, whereas in wild type animals no such labeling is visible (data not shown). The PVN is one of the most complex and heterogeneously organized nuclei of the brain; its major function is the secretion of diverse neuropeptides which are produced in distinct cell populations (Kadar et al., 2010; Biag et al., 2012). To reduce the experimental expense necessary for a molecular phenotyping of the WGA-immunoreactive cells, we first exploited the fact, that the PVN is not homogenously packed with neurons and those cells producing the different neuropeptides are not evenly distributed, but arranged in characteristic patterns (Biag et al., 2012). In a first approach the distribution of WGA-immunoreactive cells within the PVN was systematically analyzed (*n* = 2). By combining the anti-WGA immunohistochemistry with Nissl staining, it became evident that no WGA-immunoreactive cells were found within the periventricular part of the PVN, where the cell density is typically low (as shown on the representative sections in Figure 3). Since this part of the nucleus is occupied by the somatostatin-producing cells (Biag et al., 2012), these were considered as unlikely candidates. Cells with WGA-immunoreactivity were restricted to the remaining major portion of the PVN, where the cell density is high (Figures 3A–D). A detailed mapping revealed that at the most rostral level, the WGA-immunoreactive cells tended to cluster in the ventral part of the densely packed major portion of the PVN (Figure 3A). At the mid rostro-caudal extent they were located predominantly in the dorsal half (Figure 3B) and at the most caudal level in the dorsolateral part (Figures 3C and D). This particular pattern within the PVN is most reminiscent to the previously described distribution of cells producing either AVP or oxytocin (Oxy) (Biag et al., 2012). We therefore concluded that WGA-positive cells in the PVN may represent vasopressinergic, oxytonergic, or a mixture of both cell types. To scrutinize this hypothesis, double labeling experiments using the anti-WGA antibody in combination with antibodies against the two peptides were performed. Distinct populations of oxytonergic (Figure 4A) or vasopressinergic (Figure 4D) cells could be visualized by this approach. On the same sections multiple WGA-positive cells could be observed (Figures 4B and E). An overlay of these stainings demonstrates that with the anti-oxytocin antibody hardly any of these cells was double labeled (Figure 4C). In contrast, most (95.4% ± 1.4%; *n* = 3; mean ± standard deviation) of the AVP-producing cells were double labeled using the anti-WGA antibody (Figure 4F). These results showed that cells in the PVN which are labeled by the transsynaptic marker are vasopressinergic, but not oxytonergic. Previous studies have shown, that also the SO of the hypothalamus is composed of AVP- and Oxy-producing cell populations (Sabatier et al., 2003; Ludwig et al., 2005), raising the question, to which cell type in this nucleus the connectivity from the OR37C glomerulus is formed. The staining with specific antibodies against Oxy or AVP, respectively, visualized distinct populations in the SO (Figures 5A and D). On the same sections numerous WGA-positive cells could be visualized (Figures 5B and E). An overlay of the anti-WGA and the anti-oxytocin immunoreactivity (Figure 5C) revealed hardly any double labeled cells. In contrast, the AVP- and WGA-immunoreactivity almost completely overlapped (Figure 5F). These results thus showed that also in the SO, the transsynaptic marker labeled vasopressinergic cells, whereas oxytonergic cells were not stained. Thus, the connections from the OR37C glomerulus are formed to cells producing AVP in both nuclei.

**Figure 6:**
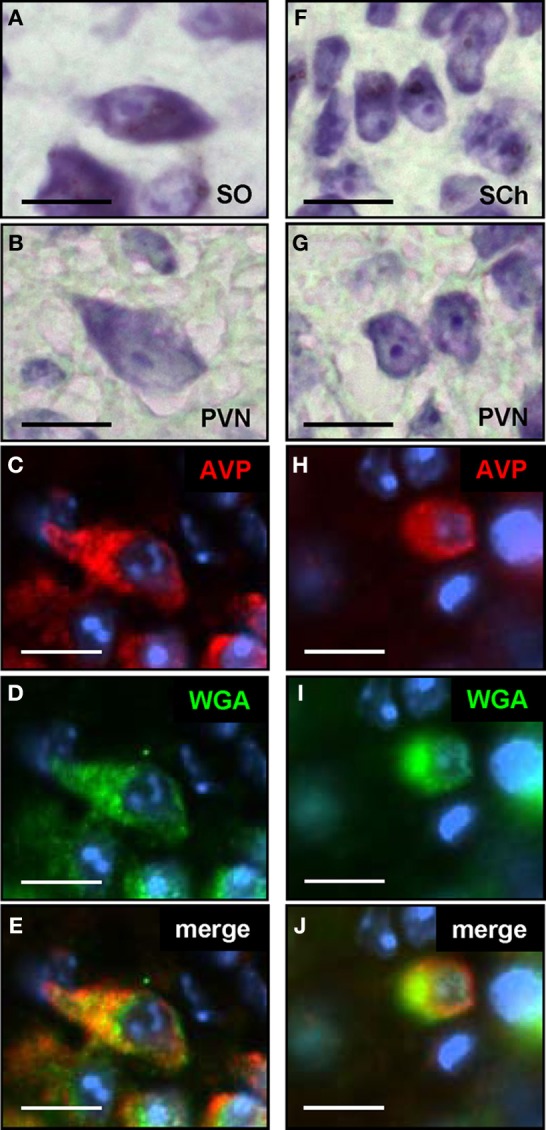
**Magno- and parvocellular vasopressinergic neurons in the paraventricular nucleus are labeled by the transsynaptic marker. (A)** Nissl staining of a section through the supraoptic nucleus (SO) reveals the typical morphology of magnocellular neurons; these cells are large in diameter and exhibit a spindle-shaped morphology. **(B)** On Nissl stained sections through the paraventricular nucleus (PVN), cells with a similar morphology as shown in **(A)**, thus magnocellular neurons, can be identified. **(C)** Section through the paraventricular nucleus after incubation with an anti-AVP antibody; neurons with a typical magnocellular morphology are stained **(D)** Section shown in **(C)** after incubation with an anti-WGA antibody. **(E)** The overlay of pictures **(C)** and **(D)** shows that this magnocellular vasopressinergic neuron is also labeled by the transsynaptic marker. **(F)** Nissl staining of a section through the suprachiasmatic nucleus (SCh) reveals the typical morphology of parvocellular neurons; these cells have a small diameter and are round-shaped. **(G)** On Nissl stained sections through the paraventricular nucleus (PVN), cells with a similar morphology as shown in **(F)**, thus parvocellular neurons, can be identified. **(H)** Section through the paraventricular nucleus after incubation with an anti-AVP antibody; neurons with a typical parvocellular morphology are stained. **(I)** Section shown in **(H)** after incubation with an anti-WGA antibody. **(J)** The overlay of pictures **(H)** and **(I)** shows that this parvocellular vasopressinergic neuron is also labeled by the transsynaptic marker. All pictures represent wide field images. Scale bars: 10 μm

Previous studies have shown that vasopressinergic cells in the SO represent a homogenous group of large, so-called magnocellular neurons (Castel and Morris, 1988), which project to the posterior pituitary (Swanson and Sawchenko, 1983). In contrast, the AVP-producing cell population in the PVN is comprised not only of magnocellular neurons, which also project to the posterior pituitary, but also of parvocellular neurons with a smaller diameter which project to multiple extra-hypothalamic sites (Sawchenko and Swanson, 1982; Antoni, 1993; Hallbeck and Blomqvist, 1999). To address the question, to which of these vasopressinergic cell types in the PVN the connectivity is formed, a comparative morphological examination of the cells was performed. To determine how the morphology of the two cell types appears in our preparations, cells from the SO which is comprised exclusively of magnocellular neurons and the suprachiasmatic nucleus (SCh) which contains only parvocellular neurons (Sofroniew and Weindl, 1978) were examined. On Nissl stained sections through the SO, the magnocellular neurons appeared as large and spindle-shaped cells, (Figure 6A) and the parvocellular neurons in the SCh were smaller and mostly round-shaped (Figure 6F). On the Nissl stained sections through the PVN, neurons with a magnocellular (Figure 6B) and with a parvocellular (Figure 6G) appearance could be identified. Based on these features, also the AVP-positive neurons in the PVN could be categorized into larger magnocellular (Figure 6C) and smaller parvocellular (Figure 6H) cells. Double labeling experiments together with the anti-WGA antibody (Figures 6D and I) revealed that cells labeled by the transsynaptic marker (Figures 6E and J) are magnocellular and parvocellular vasopressinergic neurons.

## Discussion

In the present study we have demonstrated that an application of the tracer molecule DiI onto an OR37 glomerulus on the ventral side of the MOB results in the labeling of fibers within distinct hypothalamic nuclei, the PVN and the SO. When the lipophilic dye molecule becomes inserted into the plasma membrane of a given cell, it spreads within the processes of this particular cell (Godement et al., 1987; Honig and Hume, 1989); this strongly suggests that a direct cellular connection exists between the ventral domain of the MOB and these hypothalamic nuclei. We anticipate that after the application of the dye onto an OR37 glomerulus it comes into contact with the dendritic processes of the bulbar projection neurons which are positioned at that site (Shepherd, 1972) and distributes along their axonal connections into the labeled brain areas. Based on our observations that the transsynaptic tracer WGA, which is specifically produced in OR37C expressing OSNs, arrives within the same brain areas, we conclude that projection neurons originating from this particular glomerulus send their axons into the identified hypothalamic nuclei and that this connectivity is in fact monosynaptic. Since DiI would not only label the processes of cells which project toward those brain areas, but also those of cells which project to the application site, it is principally possible that this approach also stained centrifugal neurons that support efferent input to the olfactory bulb (Davis et al., 1978; De Olmos et al., 1978). However, the fact that we did not observe DiI-labeled cell bodies within these particular brain nuclei and, moreover, no centrifugal input from there into the MOB has been observed so far, argues against the idea that centrifugal neurons were visualized in this case.

In contrast to the DiI-tracing approach which revealed labeled fibers at the PVN and the SO, the WGA-based technique stained almost exclusively cell bodies in the same brain regions. This difference is most likely due to the different properties of the labels. Whereas, DiI is lipophilic and integrates into the cell membrane of the projection neurons and thus labels their axons (Godement et al., 1987; Honig and Hume, 1989), WGA is transferred into the synaptically coupled cells, thereby labeling also their cell body (Yoshihara et al., 1999). Unfortunately, we could not visualize the fibers of the projection neurons by the WGA approach, although WGA must have been transported along their axons to reach that site in the brain. The reason for this is currently not known; it seems conceivable that most of the protein is transferred to the postsynaptic cells, leaving too little material in the incoming axons to be detectable by the applied methods.

However, both approaches together strongly support the concept that projection neurons from OR37 glomeruli send their axons directly to the PVN and the SO of the hypothalamus. Interestingly, in the rat a direct projection of neurons from the MOB to the SO has already been described (Smithson et al., 1989). In these studies this neuronal connection was visible after injection of the anterograde tracer *Phaseolus vulgaris* leucoagglutinin into the MOB and confirmed by the retrograde transport of rhodamine-labeled latex microspheres or the tracer Fluoro-Gold into the MOB after injection into the SO. Electrophysiological experiments finally supported the concept of a monosynaptic input to the SO from the MOB (Yang et al., 1995). However, at that time the labeling could not be attributed to a particular OR population. No evidence for a direct connection from the MOB to the PVN was found in previous experiments (Smithson et al., 1989). The difference to our results in the mouse could either be due to technical reasons, for example differences of the tracers that were employed. Alternatively, they could be due to species differences (rat vs. mouse); it is well-established by now that although these two species are quite related, substantial differences exist in the organization of their nervous systems (Dorn et al., 1981; Hirst et al., 2003; Biag et al., 2012).

It is well-known that as an important central regulator of the endocrine system the hypothalamus receives chemosensory information which modulates for example distinct endocrine responses related to reproduction (Schally, 1980; Kalra and Kalra, 1983). In fact, the different chemosensory organs in the nose transmit odor information to the hypothalamus, however, the currently described neuronal pathways are all multisynaptic. They include input from the accessory olfactory bulb, from where projection neurons send their axons first to the Me, which in turn projects to distinct hypothalamic nuclei (Krettek and Price, 1978; Kevetter and Winans, 1981; von Campenhausen and Mori, 2000; Salazar and Brennan, 2001; Choi et al., 2005). Also the MOS sends information via distinct regions of the olfactory cortex, such as the anterior olfactory nucleus, the piriform cortex, and the olfactory tubercle (Price et al., 1991). There is even a convergence of accessory and main olfactory pathways at the level of the Me which is further relayed to the hypothalamus (Pro-Sistiaga et al., 2007; Kang et al., 2009). In contrast to these known pathways, we now provide evidence for the existence of projection neurons from the OR37 glomeruli in the MOB which establish a direct connection to the hypothalamus. It seems conceivable that signals carried by such a monosynaptic pathway may be less subject to modification and would thereby enable these projection neurons to gain unmodified access to the hypothalamus.

Our molecular phenotyping approaches of the WGA-labeled cells in the hypothalamus revealed a rather defined neuroendocrine cell population; interestingly, in both hypothalamic nuclei, vasopressinergic cells were stained by the transsynaptic marker. These results suggest that OR37 related odor information may affect the activity of these particular cell populations. The consequence of this influence is currently unknown, however, meanwhile it is well established that vasopressinergic cells in these hypothalamic nuclei not only secrete the peptide at the posterior pituitary for osmoregulation, but also release it within the central nervous system, either at axon terminals of the vasopressinergic cells that project to distinct extra-hypothalamic sites (Sofroniew, 1980; Sawchenko and Swanson, 1982; Castel and Morris, 1988; Rood and De Vries, 2011), or at their somata and dendrites (Landgraf et al., 1995; Ludwig et al., 2005), from where it diffuses into other brain regions. Interestingly, many studies have demonstrated that centrally released vasopressin is crucially involved in the regulation of distinct social behaviors (Bielsky and Young, 2004; Caldwell et al., 2008; Albers, 2012). Consistent with these findings, the vasopressin receptor types Avpr1a and Avpr1b are found in relevant brain areas (Ostrowski et al., 1994; Vaccari et al., 1998) and it was shown that both receptors mediate effects of vasopressin on social behavior (Bielsky et al., 2004; Stevenson and Caldwell, 2012). Studies performed with Avpr1b-knock-out mice showed for example that male and female animals were less aggressive in a specific social context and exhibit deficits in their olfactory-based motivation to interact with social stimuli, although they displayed no alterations in their ability to discriminate distinct odors (Stevenson and Caldwell, 2012). In rodents, social behavior is highly dependent on olfactory cues; therefore it is plausible that at the level of the central nervous system, vasopressin is involved in the regulation of odor-induced social behaviors. Our finding of a connectivity from OR37 expressing sensory neurons in the nose to vasopressinergic cells in the hypothalamus raise the possibility that odor information detected by these receptors is involved in the regulation of social behaviors. This idea is supported by our previous findings that OR37 projection neurons also terminate in the Me (Bader et al., 2012), which receives chemosensory information from the vomeronasal organ, which has long been known to be involved in the detection of social odor cues [reviewed in Halpern and Martinez-Marcos (2003)]. The convergence of OR37 projection neurons with the accessory pathway in the medial amygdala thus supports the concept of the OR37 receptors being involved in the regulation of social behavior. Interestingly, there is also some evidence that peripheral vasopressin could play a role in mediating complex social behaviors. In a very recent study it was shown that in cichlid fish dominant males have lower amounts of the fish-homolog vasotocin in their pituitary and therefore produce more urine compared to subordinate males; it was proposed that vasotocin plays a key role in orchestrating different social phenotypes (Almeida et al., 2012). Like in fish, also in mice urine is an important mediator of social odor cues, therefore it seems possible that vasopressin couples social odor information at the level of the central nervous system to related physiological changes in the periphery.

### Conflict of interest statement

The authors declare that the research was conducted in the absence of any commercial or financial relationships that could be construed as a potential conflict of interest.

## References

[B1] Albers H. E. (2012). The regulation of social recognition, social communication and aggression: vasopressin in the social behavior neural network. Horm. Behav. 61, 283–292 10.1016/j.yhbeh.2011.10.007 22079778

[B2] Almeida O. Gozdowska M. Kulczykowska E. Oliveira R. F. (2012). Brain levels of arginine-vasotocin and isotocin in dominant and subordinate males of a cichlid fish. Horm. Behav. 61, 212–217 10.1016/j.yhbeh.2011.12.008 22206822

[B3] Antoni F. A. (1993). Vasopressinergic control of pituitary adrenocorticotropin secretion comes of age. Front. Neuroendocrinol. 14:4 10.1006/frne.1993.1004 8387436

[B4] Bader A. Breer H. Strotmann J. (2012). Untypical connectivity from olfactory sensory neurons expressing OR37 into higher brain centers visualized by genetic tracing. Histochem. Cell Biol. 137, 615–628 10.1007/s00418-012-0919-2 22294261

[B5] Bautze V. Bär R. Fissler B. Trapp M. Schmidt D. Beifuss U. (2012). Mammalian-specific OR37 receptors are differentially activated by distinct odorous fatty aldehydes. Chem. Senses 37, 479–493 10.1093/chemse/bjr130 22302156

[B6] Biag J. Huang Y. Gou L. Hintiryan H. Askarinam A. Hahn J. D. (2012). Cyto- and chemoarchitecture of the hypothalamic paraventricular nucleus in the C57BL/6J male mouse: a study of immunostaining and multiple fluorescent tract tracing. J. Comp. Neurol. 520, 6–33 10.1002/cne.22698 21674499 PMC4104804

[B7] Bielsky I. F. Hu S. B. Szegda K. L. Westphal H. Young L. J. (2004). Profound impairment in social recognition and reduction in anxiety-like behavior in vasopressin V1a receptor knockout mice. Neuropsychopharmacology 29, 483–493 10.1038/sj.npp.1300360 14647484

[B8] Bielsky I. F. Young L. J. (2004). Oxytocin, vasopressin, and social recognition in mammals. Peptides 25, 1565–1574 10.1016/j.peptides.2004.05.019 15374658

[B9] Breer H. Fleischer J. Strotmann J. (2006). The sense of smell: multiple olfactory subsystems. Cell. Mol. Life Sci. 63, 1465–1475 10.1007/s00018-006-6108-5 16732429 PMC11136015

[B10] Caldwell H. K. Lee H. J. Macbeth A. H. Young W. S. 3rd. (2008). Vasopressin: behavioral roles of an “original” neuropeptide. Prog. Neurobiol. 84, 1–24 10.1016/j.pneurobio.2007.10.007 18053631 PMC2292122

[B11] Castel M. Morris J. F. (1988). The neurophysin-containing innervation of the forebrain of the mouse. Neuroscience 24, 937–966 3380308 10.1016/0306-4522(88)90078-4

[B12] Choi G. B. Dong H. W. Murphy A. J. Valenzuela D. M. Yancopoulos G. D. Swanson L. W. (2005). Lhx6 delineates a pathway mediating innate reproductive behaviors from the amygdala to the hypothalamus. Neuron 46, 647–660 10.1016/j.neuron.2005.04.011 15944132

[B13] Davis B. J. Macrides F. Youngs W. M. Schneider S. P. Rosene D. L. (1978). Efferents and centrifugal afferents of the main and accessory olfactory bulbs in the hamster. Brain Res. Bull. 3, 59–72 10.1016/0361-9230(78)90062-X 75756

[B14] De Olmos J. Hardy H. Heimer L. (1978). The afferent connections of the main and the accessory olfactory bulb formations in the rat: an experimental HRP-study. J. Comp. Neurol. 181, 213–244 10.1002/cne.901810202 690266

[B15] Dorn A. Bernstein H. G. Hahn H. J. Ziegler M. Rummelfanger H. (1981). Insulin immunohistochemistry of rodent CNS: apparent species differences but good correlation with radioimmunological data. Histochemistry 71, 609–616 7021484 10.1007/BF00508386

[B16] Godement P. Vanselow J. Thanos S. Bonhoeffer F. (1987). A study in developing visual systems with a new method of staining neurones and their processes in fixed tissue. Development 101, 697–713 2460302 10.1242/dev.101.4.697

[B17] Hallbeck M. Blomqvist A. (1999). Spinal cord-projecting vasopressinergic neurons in the rat paraventricular hypothalamus. J. Comp. Neurol. 411, 201–211 10.1002/(SICI)1096-9861(19990823)411:2<201::AID-CNE3>3.0.CO;2-3 10404248

[B18] Halpern M. Martinez-Marcos A. (2003). Structure and function of the vomeronasal system: an update. Prog. Neurobiol. 70, 245–318 10.1016/S0301-0082(03)00103-5 12951145

[B19] Hirst W. D. Abrahamsen B. Blaney F. E. Calver A. R. Aloj L. Price G. W. (2003). Differences in the central nervous system distribution and pharmacology of the mouse 5-hydroxytryptamine-6 receptor compared with rat and human receptors investigated by radioligand binding, site-directed mutagenesis, and molecular modeling. Mol. Pharmacol. 64, 1295–1308 10.1124/mol.64.6.1295 14645659

[B20] Honig M. G. Hume R. I. (1989). Dil and diO: versatile fluorescent dyes for neuronal labelling and pathway tracing. Trends Neurosci. 12, 333–335; 340–341. 2480673

[B21] Hoppe R. Lambert T. D. Samollow P. B. Breer H. Strotmann J. (2006). Evolution of the “OR37” subfamily of olfactory receptors: a cross-species comparison. J. Mol. Evol. 62, 460–472 10.1007/s00239-005-0093-4 16547640

[B22] Imai T. Sakano H. Vosshall L. B. (2010). Topographic mapping – the olfactory system. Cold Spring Harb. Perspect. Biol. 2, a001776 10.1101/cshperspect.a001776 20554703 PMC2908763

[B23] Kadar A. Sanchez E. Wittmann G. Singru P. S. Fuzesi T. Marsili A. (2010). Distribution of hypophysiotropic thyrotropin-releasing hormone (TRH)-synthesizing neurons in the hypothalamic paraventricular nucleus of the mouse. J. Comp. Neurol. 518, 3948–3961 10.1002/cne.22432 20737594 PMC2932658

[B24] Kalra S. P. Kalra P. S. (1983). Neural regulation of luteinizing hormone secretion in the rat. Endocr. Rev. 4, 311–351 10.1210/edrv-4-4-311 6360674

[B25] Kang N. Baum M. J. Cherry J. A. (2009). A direct main olfactory bulb projection to the ‘vomeronasal’ amygdala in female mice selectively responds to volatile pheromones from males. Eur. J. Neurosci. 29, 624–634 10.1111/j.1460-9568.2009.06638.x 19187265 PMC2669936

[B26] Kevetter G. A. Winans S. S. (1981). Connections of the corticomedial amygdala in the golden hamster. I. Efferents of the “vomeronasal amygdala”. J. Comp. Neurol. 197, 81–98 10.1002/cne.901970107 6164702

[B27] Krettek J. E. Price J. L. (1978). Amygdaloid projections to subcortical structures within the basal forebrain and brainstem in the rat and cat. J. Comp. Neurol. 178, 225–254 10.1002/cne.901780204 627625

[B28] Kubick S. Strotmann J. Andreini I. Breer H. (1997). Subfamily of olfactory receptors characterized by unique structural features and expression patterns. J. Neurochem. 69, 465–475 10.1046/j.1471-4159.1997.69020465.x 9231704

[B29] Landgraf R. Neumann I. Holsboer F. Pittman Q. J. (1995). Interleukin-1 beta stimulates both central and peripheral release of vasopressin and oxytocin in the rat. Eur. J. Neurosci. 7, 592–598 7620610 10.1111/j.1460-9568.1995.tb00663.x

[B30] Lin W. Margolskee R. Donnert G. Hell S. W. Restrepo D. (2007). Olfactory neurons expressing transient receptor potential channel M5 (TRPM5) are involved in sensing semiochemicals. Proc. Natl. Acad. Sci. U.S.A. 104, 2471–2476 10.1073/pnas.0610201104 17267604 PMC1892929

[B31] Ludwig M. Bull P. M. Tobin V. A. Sabatier N. Landgraf R. Dayanithi G. (2005). Regulation of activity-dependent dendritic vasopressin release from rat supraoptic neurones. J. Physiol. 564, 515–522 10.1113/jphysiol.2005.083931 15731188 PMC1464450

[B32] Ma M. (2007). Encoding olfactory signals via multiple chemosensory systems. Crit. Rev. Biochem. Mol. Biol. 42, 463–480 10.1080/10409230701693359 18066954

[B33] Mori K. Sakano H. (2011). How is the olfactory map formed and interpreted in the mammalian brain? Annu. Rev. Neurosci. 34, 467–499 10.1146/annurev-neuro-112210-112917 21469960

[B34] Munger S. D. Leinders-Zufall T. Zufall F. (2009). Subsystem organization of the mammalian sense of smell. Annu. Rev. Physiol. 71, 115–140 10.1146/annurev.physiol.70.113006.100608 18808328

[B35] Murthy V. N. (2011). Olfactory maps in the brain. Annu. Rev. Neurosci. 34, 233–258 10.1146/annurev-neuro-061010-113738 21692659

[B36] Ostrowski N. L. Lolait S. J. Young W. S. 3rd. (1994). Cellular localization of vasopressin V1a receptor messenger ribonucleic acid in adult male rat brain, pineal, and brain vasculature. Endocrinology 135, 1511–1528 10.1210/en.135.4.1511 7925112

[B37] Paxinos G. Franklin K. B. J. (2001). The Mouse Brain in Stereotaxic Coordinates. San Diego, CA: Academic Press

[B38] Price J. L. Slotnick B. M. Revial M. F. (1991). Olfactory projections to the hypothalamus. J. Comp. Neurol. 306, 447–461 10.1002/cne.903060309 1713925

[B39] Pro-Sistiaga P. Mohedano-Moriano A. Ubeda-Banon I. Mar Arroyo-Jimenez M. Marcos P. Artacho-Perula E. (2007). Convergence of olfactory and vomeronasal projections in the rat basal telencephalon. J. Comp. Neurol. 504, 346–362 10.1002/cne.21455 17663431

[B40] Rood B. D. De Vries G. J. (2011). Vasopressin innervation of the mouse (*Mus musculus*) brain and spinal cord. J. Comp. Neurol. 519, 2434–2474 10.1002/cne.22635 21456024 PMC3939019

[B41] Sabatier N. Caquineau C. Dayanithi G. Bull P. Douglas A. J. Guan X. M. (2003). Alpha-melanocyte-stimulating hormone stimulates oxytocin release from the dendrites of hypothalamic neurons while inhibiting oxytocin release from their terminals in the neurohypophysis. J. Neurosci. 23, 10351–10358 14614094 10.1523/JNEUROSCI.23-32-10351.2003 PMC6741015

[B42] Sakano H. (2010). Neural map formation in the mouse olfactory system. Neuron 67, 530–542 10.1016/j.neuron.2010.07.003 20797531

[B43] Salazar I. Brennan P. A. (2001). Retrograde labelling of mitral/tufted cells in the mouse accessory olfactory bulb following local injections of the lipophilic tracer DiI into the vomeronasal amygdala. Brain Res. 896, 198–203 10.1016/S0006-8993(01)02225-9 11277993

[B44] Sawchenko P. E. Swanson L. W. (1982). Immunohistochemical identification of neurons in the paraventricular nucleus of the hypothalamus that project to the medulla or to the spinal cord in the rat. J. Comp. Neurol. 205, 260–272 10.1002/cne.902050306 6122696

[B45] Schaefer M. L. Yamazaki K. Osada K. Restrepo D. Beauchamp G. K. (2002). Olfactory fingerprints for major histocompatibility complex-determined body odors II: relationship among odor maps, genetics, odor composition, and behavior. J. Neurosci. 22, 9513–9521 12417675 10.1523/JNEUROSCI.22-21-09513.2002 PMC6758037

[B46] Schaefer M. L. Young D. A. Restrepo D. (2001). Olfactory fingerprints for major histocompatibility complex-determined body odors. J. Neurosci. 21, 2481–2487 11264322 10.1523/JNEUROSCI.21-07-02481.2001 PMC6762408

[B47] Schally A. V. (1980). Aspects of hypothalamic regulation of the pituitary gland. Its implications for the control of reproductive processes. Mater. Med. Pol. 12, 9–27 6120267

[B48] Shepherd G. M. (1972). Synaptic organization of the mammalian olfactory bulb. Physiol. Rev. 52, 864–917 4343762 10.1152/physrev.1972.52.4.864

[B49] Smithson K. G. Weiss M. L. Hatton G. I. (1989). Supraoptic nucleus afferents from the main olfactory bulb–I. Anatomical evidence from anterograde and retrograde tracers in rat. Neuroscience 31, 277–287 10.1016/0306-4522(89)90373-4 2477769

[B50] Sofroniew M. V. (1980). Projections from vasopressin, oxytocin, and neurophysin neurons to neural targets in the rat and human. J. Histochem. Cytochem. 28, 475–478 10.1177/28.5.7381192 7381192

[B51] Sofroniew M. V. Weindl A. (1978). Projections from the parvocellular vasopressin- and neurophysin-containing neurons of the suprachiasmatic nucleus. Am. J. Anat. 153, 391–429 10.1002/aja.1001530305 360814

[B52] Stevenson E. L. Caldwell H. K. (2012). The vasopressin 1b receptor and the neural regulation of social behavior. Horm. Behav. 61, 277–282 10.1016/j.yhbeh.2011.11.009 22178035 PMC3310934

[B53] Strotmann J. Conzelmann S. Beck A. Feinstein P. Breer H. Mombaerts P. (2000). Local permutations in the glomerular array of the mouse olfactory bulb. J. Neurosci. 20, 6927–6938 10995837 10.1523/JNEUROSCI.20-18-06927.2000 PMC6772838

[B54] Strotmann J. Wanner I. Krieger J. Raming K. Breer H. (1992). Expression of odorant receptors in spatially restricted subsets of chemosensory neurones. Neuroreport 3, 1053–1056 1493216 10.1097/00001756-199212000-00005

[B55] Swanson L. W. Sawchenko P. E. (1983). Hypothalamic integration: organization of the paraventricular and supraoptic nuclei. Annu. Rev. Neurosci. 6, 269–324 10.1146/annurev.ne.06.030183.001413 6132586

[B56] Vaccari C. Lolait S. J. Ostrowski N. L. (1998). Comparative distribution of vasopressin V1b and oxytocin receptor messenger ribonucleic acids in brain. Endocrinology 139, 5015–5033 10.1210/en.139.12.5015 9832441

[B57] von Campenhausen H. Mori K. (2000). Convergence of segregated pheromonal pathways from the accessory olfactory bulb to the cortex in the mouse. Eur. J. Neurosci. 12, 33–46 10.1046/j.1460-9568.2000.00879.x 10651858

[B58] Yang Q. Z. Smithson K. G. Hatton G. I. (1995). NMDA and non-NMDA receptors on rat supraoptic nucleus neurons activated monosynaptically by olfactory afferents. Brain Res. 680, 207–216 10.1016/0006-8993(95)00153-H 7663978

[B59] Yoshihara Y. Mizuno T. Nakahira M. Kawasaki M. Watanabe Y. Kagamiyama H. (1999). A genetic approach to visualization of multisynaptic neural pathways using plant lectin transgene. Neuron 22, 33–41 10.1016/S0896-6273(00)80676-5 10027287

